# Collaboration of tRNA modifications and elongation factor eEF1A in decoding and nonsense suppression

**DOI:** 10.1038/s41598-018-31158-2

**Published:** 2018-08-24

**Authors:** Roland Klassen, Raffael Schaffrath

**Affiliations:** Institut für Biologie, Fachgebiet Mikrobiologie, Universität Kassel, Heinrich-Plett-Str. 40, D-34132 Kassel, Germany

## Abstract

Transfer RNA (tRNA) from all domains of life contains multiple modified nucleosides, the functions of which remain incompletely understood. Genetic interactions between tRNA modification genes in *Saccharomyces cerevisiae* suggest that different tRNA modifications collaborate to maintain translational efficiency. Here we characterize such collaborative functions in the ochre suppressor tRNA *SUP4*. We quantified ochre read-through efficiency in mutants lacking either of the 7 known modifications in the extended anticodon stem loop (G26-C48). Absence of U34, U35, A37, U47 and C48 modifications partially impaired *SUP4* function. We systematically combined modification defects and scored additive or synergistic negative effects on *SUP4* performance. Our data reveal different degrees of functional redundancy between specific modifications, the strongest of which was demonstrated for those occurring at positions U34 and A37. *SUP4* activity in the absence of critical modifications, however, can be rescued in a gene dosage dependent fashion by *TEF1* which encodes elongation factor eEF1A required for tRNA delivery to the ribosome. Strikingly, the rescue ability of higher-than-normal eEF1A levels extends to tRNA modification defects in natural non-suppressor tRNAs suggesting that elevated eEF1A abundance can partially compensate for functional defects induced by loss of tRNA modifications.

## Introduction

Transfer RNA (tRNA) is known for the presence of extensive post-transcriptional modifications. Some of the non-standard ribonucleosides are thought to be important for tRNA stability and folding or to improve codon-anticodon recognition^1–3^. The latter has been attributed to the wobble uridine modification 5-methoxy-carbonyl-methyl-2-thiouridine (mcm^5^s^2^U)^4–7^. In yeast, this modification is naturally present in tRNA^Lys^_UUU_, tRNA^Gln^_UUG_ and tRNA^Glu^_UUC_ and is formed by two separate pathways which mediate mcm^5^ side chain addition at position 5 of the uracil base and exchange of the oxygen for sulfur at position 2 (wobble uridine thiolation)^8^. Genes required for addition of the mcm^5^ side chain include *ELP1-ELP6* (Elongator), *TRM9-TRM112* (wobble methyltransferase) and *KTI11-KTI14* or *SIT4*, other potentially regulatory loci^4,9–17^. The thiolation of tRNA^Lys^_UUU_, tRNA^Gln^_UUG_ and tRNA^Glu^_UUC_ depends on the ubiquitin like modifier Urm1 and several other sulfur transfer proteins^11,18–21^.

Several lines of evidence support a role of mcm^5^s^2^U in improvement of tRNA binding to the ribosomal A-site; in the absence of the modification, reduced A-site binding evokes downstream effects, including ribosomal slow down and associated protein folding defects as well as increased frameshift errors^5,22–24^. *In vitro* studies with bacterial ribosomes suggest that absence of s^2^U already increases tRNA rejection both before and after GTP hydrolysis by EF-Tu and impedes ribosomal translocation^7^. In all cases analysed, the combined absence of mcm^5^ and s^2^U led to more severe effects and phenotypes compared to loss of either part alone, suggesting that mcm^5^ and s^2^U are to some extent functionally redundant in maintaining tRNA decoding efficiency^22,24–26^.

The genes required for mcm^5^ and s^2^U formation form an extensive network of negative genetic interactions with loci required for other tRNA modifications, such as *DEG1* (pseudouridine, ψ38/39) and *TCD1* (cyclic N6-threonylcarbamoyadenosine, ct^6^A37)^27–29^. *In vivo* studies support the conclusion that malfunction of tRNA^Lys^_UUU_ in the combined absence of mcm^5^/s^2^U and ct^6^A and malfunction of tRNA^Gln^_UUG_ in the combined absence of mcm^5^/s^2^U and ψ38 is the underlying cause of the observed negative genetic interactions^30^. Hence, modifications at distinct positions of the anticodon loop might cooperatively promote tRNA function, an idea that is further supported by *in vitro* studies indicating an anticodon-prestructuring role shared between U34 and A37 modifications^1,2,6,26,31^. Functional redundancy between different modifications may explain why the majority of single tRNA modification defects has only mild or no negative effects on growth, whereas combined modification mutants show more severe phenotypes in several cases. However, so far, no study is available to the best of our knowledge that quantified the vivo function of a defined tRNA in the combined absence of different modifications.

We utilized in here the non-sense ochre suppressor tRNA *SUP4* to measure its translational efficiency and UAA read-through in the presence and absence of modifications. *SUP4* is a variant of tRNA^Tyr^_GψU_ with a G to U exchange at the wobble base that renders the anticodon cognate for ochre stop codons^32^. The U34 position of *SUP4* is known to be modified in an Elongator dependent manner to mcm^5^U and previously, loss of mcm^5^ was shown to severely (but not entirely) diminish nonsense suppression^4,10,33^. Apart from mcm^5^U also i^6^A37 (N6-isopentenyl-adenosine) is known to be required for efficient UAA read-through by *SUP4*^34^. Further, ψ35 formation was shown to require the presence of an intron in the tRNA^Tyr^ gene and nonsense suppression by the tyrosine inserting ochre suppressor *SUP6* depends on the presence of the intron in the *SUP6* gene^35^. Thus, it appears likely that ψ35 is important for efficient nonsense suppression by tyrosine inserting suppressor tRNAs (*SUP4* and *SUP6*) as well.

We quantified *in vivo* UAA read-through efficiency of *SUP4* and relative importance of all modifications present in the extended anticodon stem and loop (ASL) including the variable loop (VL) (G26 to C48). Systematic combination of modification defects and subsequent measurement of suppressor activity provided support for functional collaboration between distinct modifications in the entire extended ASL. The strongest functional impairment of *SUP4* was observed in the combined absence of mcm^5^U and i^6^A37 reinforcing the notion that U34 and different A37 modifications in general might fulfil overlapping roles in maintaining the anticodon open loop configuration^2,26^. Genetic interaction networks of the studied modification genes further suggested a role of eEF1A gene dosage in changing the dependency of tRNA on the presence of modifications, which was directly demonstrated for *SUP4* tRNA.

## Results

### Comparison of relative contributions of single tRNA modifications for *SUP4* functioning

**Figure 1:**
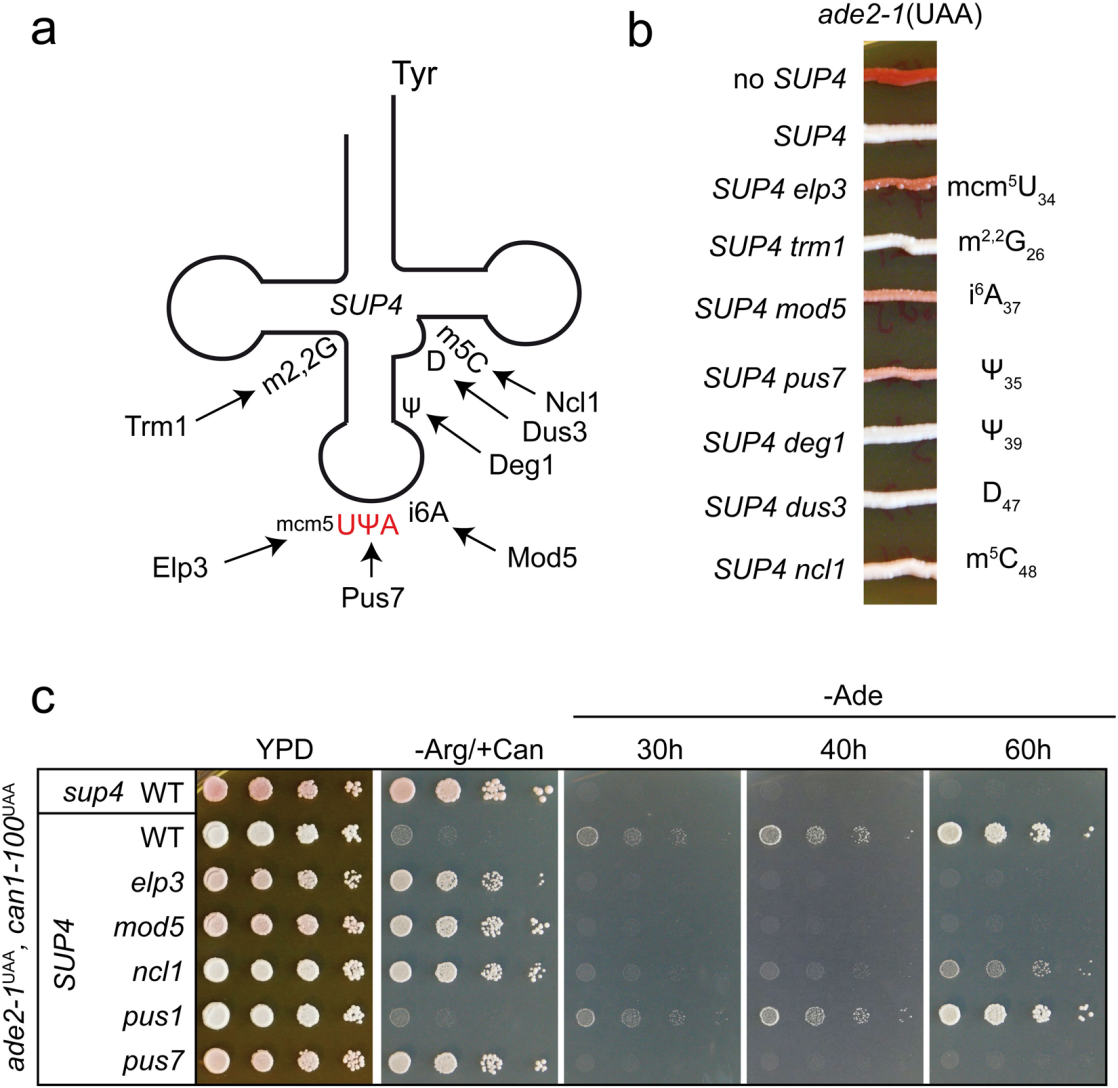
Relevance of individual tRNA modifications for ochre suppression by *SUP4*. (**a**) Schematic representation of *SUP4* tRNA. Modifications are indicated according to^36^ together with proteins essential for their formation. (**b**) Red pigmentation of yeast strains of the indicated genetic background as an indicator for *ade2-1* suppression. Strains were streaked on YPD, grown for 4 days, followed by incubation at 4 °C for an additional 4 days prior to documentation. (**c**) Plate assays of *ade2-1* and *can1-100* suppression by *SUP4*. Strains of the indicated genetic backgrounds were spotted on YPD, −Ade or −Arg/+Can media and photographed after 40 h of incubation at 30 °C (YPD and −Arg/+Can) or at the indicated time points (−Ade).

To systematically investigate the role of individual modifications in *SUP4*, we targeted all known modifications of the extended ASL spanning from position 26 to 48. In tRNA^Tyr^_GψA_, this region is reported to harbour modified nucleosides N2,N2-dimethylguanosine (m^2,2^G) at position 26, pseudouridine (ψ) at positions 35 and 39, N6-isopentenyladenosine (i^6^A) at position 37, dihydrouridine (D) at position 47 and 5-methylcytosine (m^5^C) at position 48 (Fig. 1A)^36^. In addition, the G:U exchange at position 34 renders *SUP4* an Elongator substrate carrying mcm^5^U^10^. These modifications are introduced by well characterized modifiers that can be inactivated by single gene deletions^10,37–41^. We used existing *elp3* (mcm^5^U) and *deg1* (ψ39) mutants in a W303-1B derived *SUP4* strain^10,42^ and additionally deleted *TRM1* (m^2,2^G), *PUS7* (ψ35), *MOD5* (i^6^A), *DUS3* (D47) and *NCL1* (m^5^C) to assemble a set of mutants individually defective in each of the 7 modifications of the extended ASL of *SUP4*. We also deleted *PUS1* since earlier evidence indicated an ability of recombinant Pus1 to modify U35 in pre-tRNA^Tyr^_GUA/UUA_ *in vitro*^43^. The parental strain utilized carries *ade2-1* and *can1-100* alleles containing premature UAA stop codons which can be suppressed by *SUP4*^10^. Nonsense suppression of *ade2-1* enables growth on −Ade media and suppresses red pigmentation normally associated with loss-of-function *ade2* alleles. UAA stop codon read-through of *can1-100* by functional *SUP4* results in sensitivity to the arginine analogue canavanine. As expected, the presence of *SUP4* entirely suppressed red pigmentation in the wild type and loss of *ELP3* (mcm^5^U), *MOD5* (i^6^A) and *PUS7* (ψ35) partially restored pigment formation, albeit to a lesser extent compared to the parental strain lacking *SUP4* tRNA altogether (Fig. 1B). When all single mutants were analysed for *can1-100* and *ade2-1* suppression using serial dilution spot assays on appropriate media, loss of suppression to comparable degrees of both *ade2-1* and *can1-100* was observed for *elp3*, *mod5* and *pus7* (Fig. 1C). The *ncl1* mutant defective in m^5^C formation^41^ entirely lost *can1-100* suppression and was partially defective in *ade2-1* suppression, observable due to its delayed growth on −Ade media. Absence of *DEG1*, *TRM1*, *PUS1* and *DUS3* did not detectably affect suppression of the two reporter alleles (Figs 1C, S1).

The change of pigmentation from white to pink suggested residual non-sense suppression in *elp3*, *mod5* and *pus7* mutants since the complete absence of *SUP4* resulted in much stronger red pigmentation (Fig. 1B). *SUP4* nonsense suppression was previously quantified using *lacZ* reporters with a premature UAA codon and indeed significant residual nonsense suppression in *elp3* mutants was observed, suggesting that canavanine resistance and inability of modification mutant strains to grow on −Ade media does not necessarily indicate a complete loss of *SUP4* function^4,42^.

### *SUP4* dependent nonsense suppression in the absence of nonsense mediated decay

**Figure 2:**
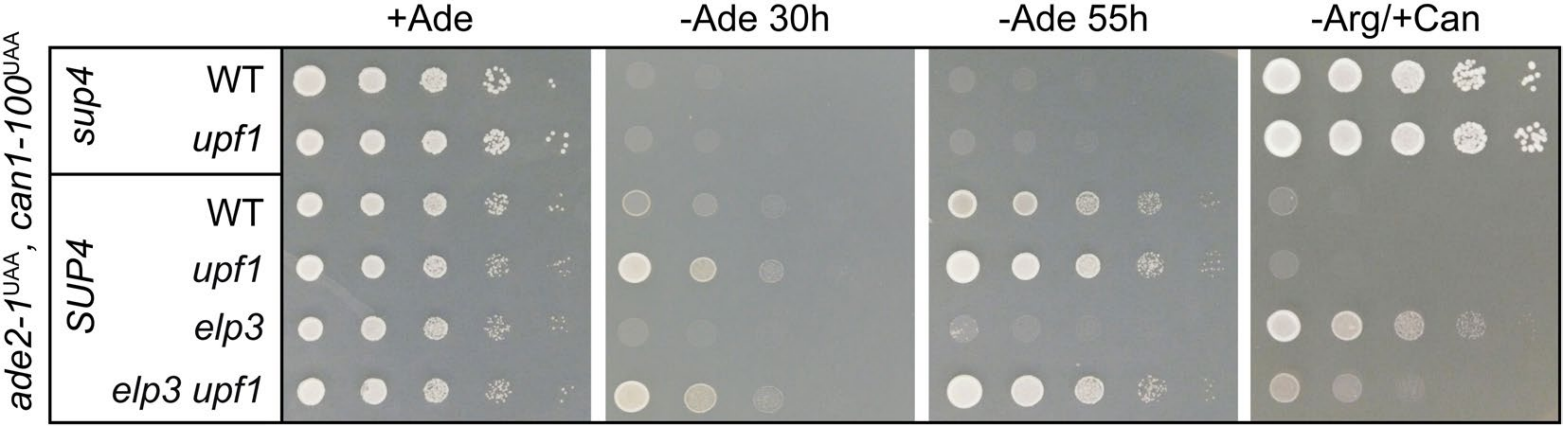
*SUP4* dependent ochre suppression in absence of nonsense mediated decay. Plate assays of *ade2-1* and *can1-100* suppression by *SUP4*. Strains of the indicated genetic backgrounds were spotted on +Ade, −Ade or −Arg/+Can media and photographed after 40 h of incubation at 30 °C (+Ade and −Arg/+Can) or at the indicated time points (−Ade).

Since *ade2-1* and *can1-100* nonsense mRNAs are drastically stabilized in the absence of nonsense mediated decay (NMD)^44,45^, we investigated *SUP4* dependent nonsense suppression in the absence of a functional NMD pathway. Because nonsense suppressor tRNA is in competition with translation termination factors^46^, which also interact with Upf1 to initiate NMD of transcripts with premature termination codons^47,48^, it appeared possible that *SUP4* may affect nonsense suppression indirectly by inhibiting NMD. To test the role of NMD in *SUP4* readouts used in this study, we deleted the *UPF1* gene essential for decay of *can1-100* and *ade2-1* mRNAs^44,45^ in wild type and *SUP4* strains and scored phenotypic suppression of both reporter genes. Deletion of *UPF1* did not enable growth on −Ade media or suppress canavanine resistance in the absence of *SUP4* (Fig. 2). However, growth on −Ade media is detectably improved in a *SUP4 upf1* strain as compared to the *SUP4 UPF1* parent (Fig. 2), indicating that *SUP4* dependent generation of functional Ade2 protein from the nonsense transcript is improved by stabilization of the mRNA. Also, the above described defect in *SUP4* dependent suppression of *ade2-1* and *can1-100* in the absence of a functional *ELP3* gene can be bypassed by inactivation of NMD. The latter can be concluded due to the reversion of adenine auxotrophy and canavanine resistance in *SUP4 elp3* by deletion of *UPF1* (Fig. 2). Together these results indicate that *SUP4* functions independently of NMD in *ade2-1* and *can1-100* suppression and that absence of NMD improves the absolute *SUP4* dependent nonsense readthrough.

### Quantification of *SUP4* dependent readthrough using a dual luciferase reporter

**Figure 3:**
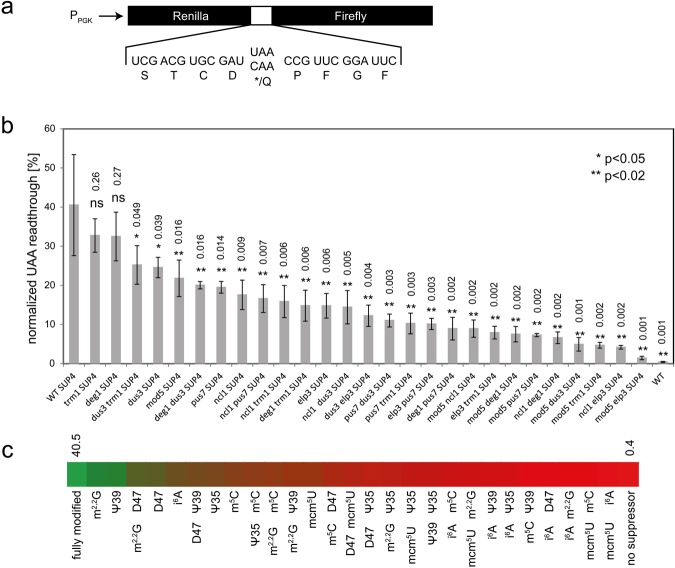
Quantification of *SUP4* mediated ochre suppression by dual luciferase assays in single and double tRNA modification mutants. (**a**) Schematic representation of dual luciferase control (pDB688) and ochre stop (pDB723) constructs. (**b**) Normalized UAA readthrough was calculated for indicated strains by dividing the renilla/firefly activity ratios of the ochre stop construct (pDB723) by the one obtained with the control construct (pDB688) multiplied by 100. All strains are sorted according to readthrough efficiency. Values significantly different from wild type (WT) *SUP4* as per two-tailed t-test are indicated (*p < 0.05; **p < 0.02). Actual p-values are indicated. (**c**) Heat bar representing *SUP4* translational efficiency (from 40.5% UAA readthrough to 0.4%) in the absence of indicated modifications.

Since we demonstrated *SUP4* to function independently of NMD but found *ade2-1* and *can1-100* suppression to be influenced by levels of the respective mRNAs, we next employed a nonsense readthrough reporter insensitive to changes in mRNA levels to quantify UAA readthrough in absence of tRNA modifications. For this purpose, we used dual luciferase constructs consisting of an upstream (renilla) and a downstream (firefly) luciferase gene, separated by a linker region containing either a sense or an in-frame nonsense (UAA) codon^49^. Since relative readthrough levels are determined based on the two reporter activities that are encoded on a single mRNA and utilizing a shared AUG start codon, they are insensitive to changes of reporter mRNA levels. Both constructs were introduced into the set of mutants and wild type control strains (carrying or lacking *SUP4*) and normalized UAA read-through levels were determined (Fig. 3). In the absence of modification defects, the presence of *SUP4* correlated with a 94-fold increase in UAA read-through levels (from 0.4 to ~40%), indicating the suitability of this assay system to quantify *SUP4* function. Comparable to results obtained with *lacZ* based reporters, loss of *ELP3* resulted in a significant drop of read-through efficiency to ~15%, equivalent to 37% of wild type level. The normalized read-through level of the mutant was significantly different (p < 0.02) from both the *SUP4* strain without modification defect and the control strain lacking *SUP4*, revealing a substantial residual read-through activity of *SUP4* in the absence of mcm^5^U. As expected from results obtained with *can1-100* and *ade2-1* alleles (Fig. 1), loss of *PUS7* (ψ35), *MOD5* (i^6^A) and *NCL1* (m^5^C) also significantly reduced *SUP4* mediated UAA read-through in the dual luciferase reporter, without abolishing it entirely (17–20% residual read-through level, p < 0.02; Fig. 3). In contrast and consistent with results obtained for *ade2-1* and *can1-100* suppression, *deg1* (ψ39) and *trm1* (m^2,2^G) mutations did not significantly lower *SUP4* activity. However, a *dus3* (D47) mutation, which did not result in detectable loss of *ade2-1* and *can1-100* suppression mildly lowered read-through in the dual luciferase construct to ~25% (p < 0.05) (Fig. 3).

### Combined *SUP4* modification loss uncovers functional redundancy

Since individual loss of m^5^C, mcm^5^U, i^6^A, ψ35 and D47 detectably lowered *SUP4* activity without abolishing it, whereas m^2,2^G and ψ39 did not appear to be important (Fig. 3), we decided to investigate the consequence of combined modification loss on *SUP4* function and performance. We attempted to generate all possible combinations of modification defects within the extended ASL to quantify the combined impact on *SUP4* activity. 20 out of 21 theoretically possible mutant combinations could be generated, all of which grew without severe defects at 30 °C (Fig. S2A). Solely the combination *elp3 deg1* was not obtained due to synthetic lethality in the W303-1B derived *SUP4* strain (Fig. S2B). We previously generated this mutant combination in a distinct strain background (S288C derived) and observed most severe growth defects and cytological abnormalities^30^. Thus, we continued the *SUP4* functional profiling with the remaining set of 20 viable double mutant combinations and quantified UAA read-through using the dual luciferase assay (Fig. 3). Double and single mutants were sorted according to their remaining *SUP4* activity. Most of the modification defects that reduced *SUP4* function in single mutants led to stronger reduction in the combination mutants, indicative of independent contributions of the modifications to *in vivo* function of the tRNA.

Residual read-through levels (UAA read-through %) in double mutants were normalized to the wild type *SUP4* strain and compared to levels of the single mutants to categorize the extent of genetic interaction between the modification genes of interest. Using the normalized read-through levels, we calculated the value expected for the respective double mutants (relative activity ∆A * relative activity ∆B) and subtracted it from the experimentally determined value for the double mutant (∆A ∆B), providing a measure of genetic interaction. When the resulting values were sorted, strong synergistic effects could be distinguished from more additive ones (Fig. S3). Strikingly, strong synergistic effects were observed in combinations involving the *deg1* and *trm1* mutations, which combine ψ39 or m^2,2^G deficiencies with other modification defects. This result indicates that m^2,2^G and ψ39, which are dispensable for *SUP4* function in the presence of all other modifications become functionally important in the absence of other modifications. Also, the combination of ψ39 and m^2,2^G deficiency led to a substantial reduction in *SUP4* function, despite the absence of detectable effects in the respective single mutants. Whereas most combinations of modification defects still allowed residual *SUP4* function (Fig. 3), a near complete loss of *SUP4* activity was observed for the *mod5 elp3* double mutant simultaneously lacking mcm^5^U and i^6^A (Fig. 3).

### Genetic interaction of *SUP4* relevant modification genes with *TEF1/TEF2*

The above data suggested a functional interaction between distinct tRNA modifications in maintaining translational efficiency of *SUP4*. Since tRNA function in translation is strictly dependent on the formation of a ternary complex between eEF1A/GTP and aminoacyl-tRNA, we investigated existing synthetic genetic array datasets^27^ for genetic interactions between eEF1A (encoded by the paralogs *TEF1* and *TEF2*) and *SUP4*-relevant tRNA modification genes. *TEF1* and *TEF2* display negative genetic interactions with 307 and 635 genes, respectively^27^. Among the strongest negative interactors, we identified several tRNA modification genes involved in mcm^5^/mcm^5^s^2^U formation (Table S2). Gene ontology (GO) analysis also revealed the GO term “tRNA modification” to be significantly enriched in *tef1/tef2* negative genetic interactors (p < 2 * 10^−5^) and identified several of the modification genes shown in here to be relevant for *SUP4* function (Elongator and interactor genes, *PUS7*, *MOD5*; Table S2). These results suggest that reduced eEF1A abundance enforces the requirement for modifications to maintain tRNA function and prompted us to investigate the functional impact of reducing eEF1A abundance on *SUP4* function. Of note, it was already demonstrated that halving the eEF1A gene copy number lowers the efficiency of a tyrosine inserting amber suppressor (*SUP7*-a)^50^. While this work in progress, it was further demonstrated that yeast phenotypes caused by loss of the tRNA methyltransferase Trm7 can be aggravated or suppressed, depending on whether eEF1A abundance is lowered or increased^51^.

**Figure 4:**
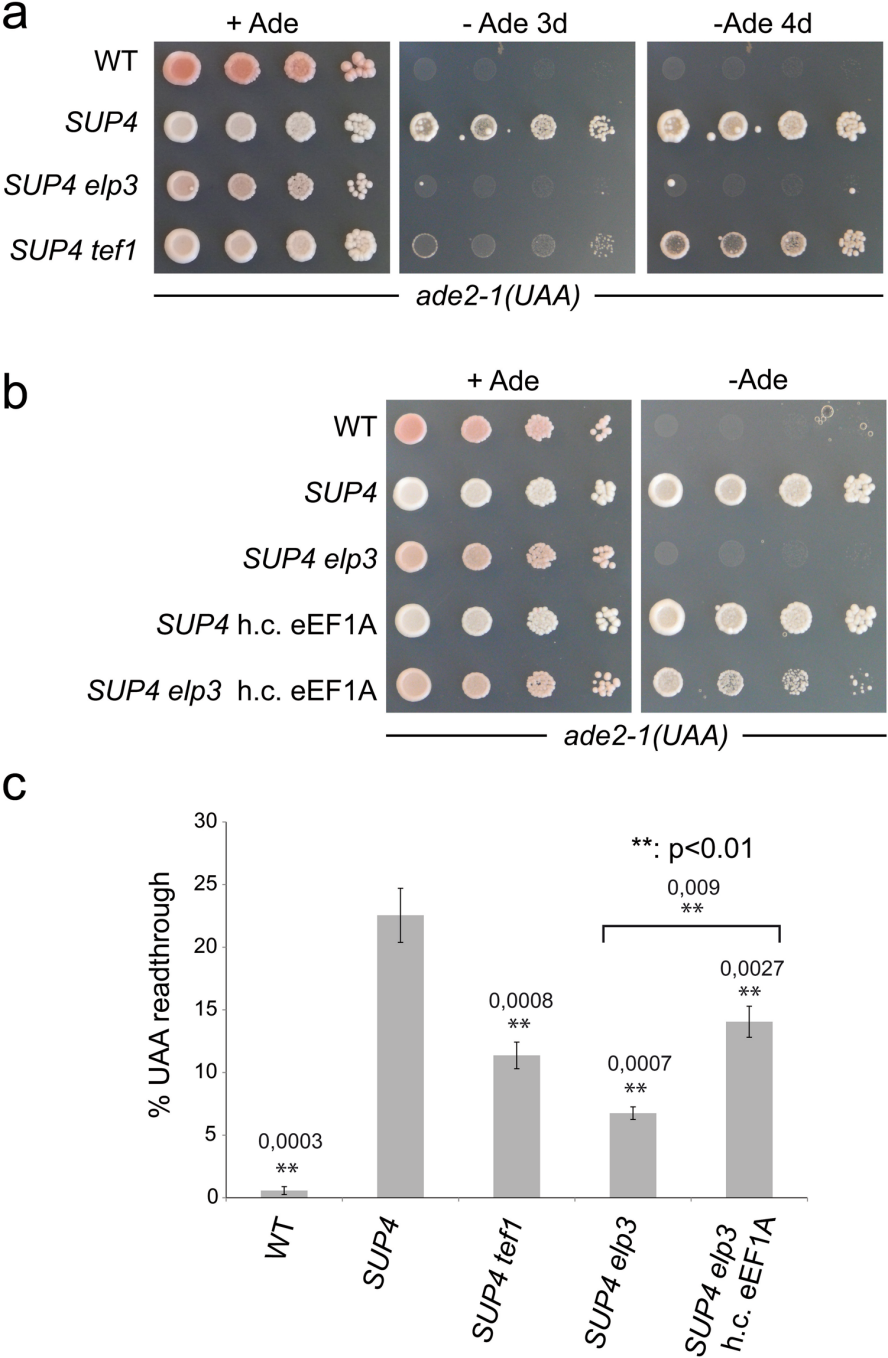
Effect of *TEF1* (eEF1A) deletion or overexpression on *SUP4* mediated ochre suppression efficiency. (**a**) Plate assays of *ade2-1* suppression in wild type (no *SUP4*), wild type *SUP4* (*SUP4*) and *SUP4 elp3* as well as *SUP4 tef1* mutants. −Ade plates were photographed after 3 and 4 days; +Ade plate was photographed after 3 days. (**b**) Plate assay of *ade2-1* suppression of indicated strains. h.c. eEF1A refers to multi-copy *TEF1* expression construct (pTEF1). −Ade plate was photographed after 3 days, −Ade plate was photographed after 4 days. (**c**) Quantification of UAA readthrough in the indicated strains using a *lacZ*-UAA and a *lacZ* control construct^42^. Values significantly different from wild type (WT) *SUP4* as per two-tailed t-test are indicated above bars (**p < 0.01). The same test revealed significant difference between *SUP4 elp3* and *SUP4 elp3* h.c. eEF1A (indicated by bracket). Actual p-values are indicated.

**Figure 5:**
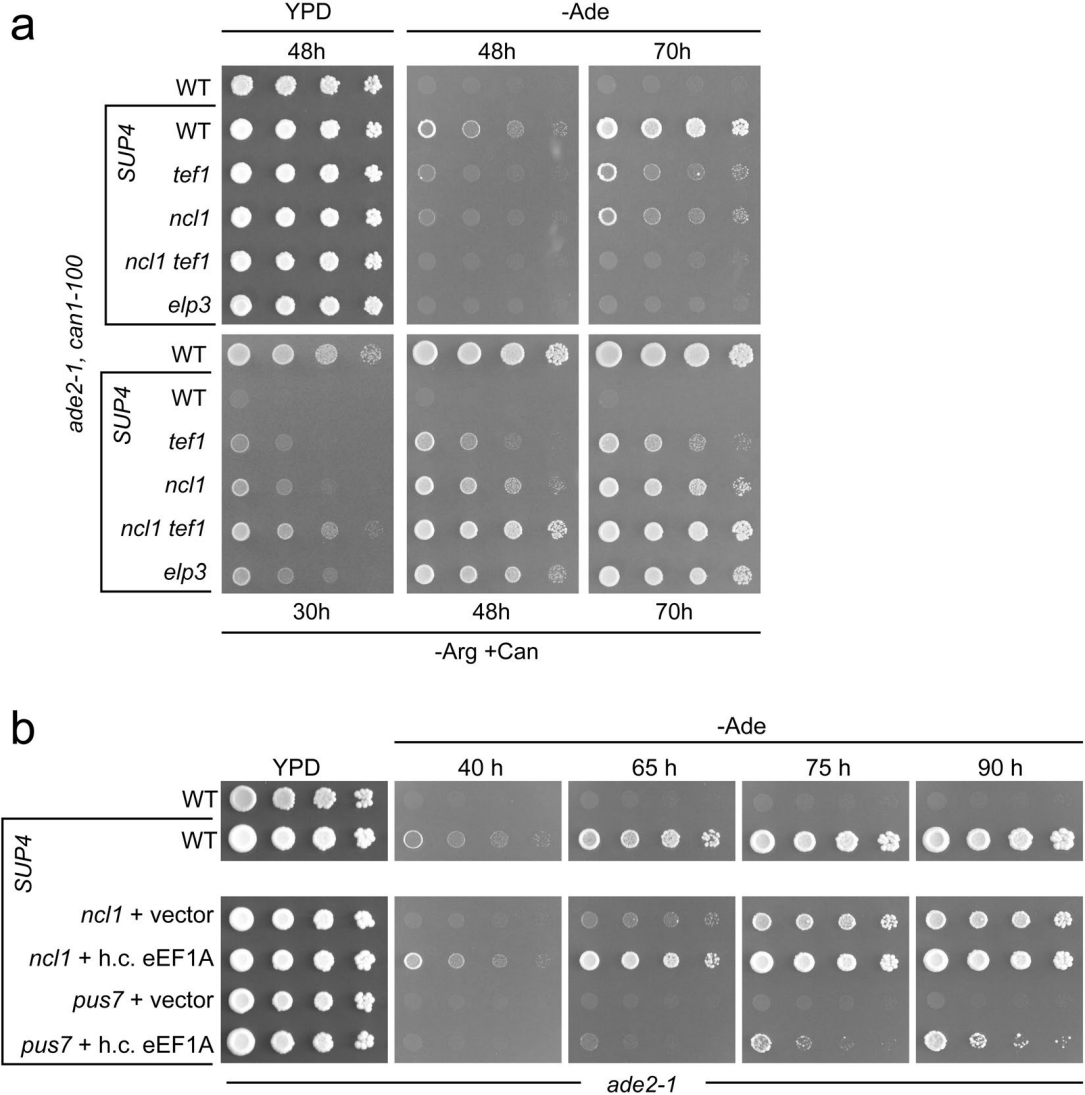
Additive *SUP4* defect in *ncl1 tef1* mutants and *SUP4* rescue efficiency of other modification mutants by overexpression of *TEF1*. (**a**) Plate assays for *ade2-1* (−Ade) and *can1-100* (−Arg/+Can) readthrough in *ncl1 tef1* mutants compared to respective single mutants. (**b**) Plate assay for *ade2-1* readthrough in indicated tRNA modification mutants carrying either vector (YEplac181) or overexpression construct for *TEF1* (h.c. eEFA, pTEF1). Plates were photographed at indicated time points. YPD plate in (B) was photographed after 48 h.

To test whether eEF1A copy number reduction also impairs the ochre suppressor *SUP4*, we deleted the *TEF1* gene in the *SUP4* strain used in this study and scored *ade2-1* read-through efficiency (Fig. 4). Indeed, *TEF1* deletion clearly impaired *ade2-1* suppression by *SUP4*. Reduced nonsense suppression in the *tef1* mutant strain was also confirmed using *lacZ* based reporter constructs^52^ carrying a premature UAA codon (Fig. 4). In both *ade2-1* and *lacZ*-UAA, *tef1* mutation lowered nonsense suppression significantly (p = 0.0008), confirming that eEF1A copy number is critical for *SUP4* mediated nonsense read-through. To test whether the partial *SUP4* defect caused by half the eEF1A copy number is additive with the negative effect caused by *SUP4* hypomodification, we used the *SUP4 ncl1* mutant that was partially defective in *ade2-1* nonsense suppression (Fig. 1) to generate a *SUP4 ncl1 tef1* double mutant. In this mutant both, *ade2-1* and *can1-100* alleles are less efficiently suppressed by *SUP4* compared to either single mutant (Fig. 5). Thus, high eEF1A levels and a complete tRNA modification set independently maintain UAA read-through capacity of *SUP4*.

### Functional rescue of hypomodified *SUP4* by elevated eEF1A levels

Since the above results suggest that eEF1A abundance and tRNA modifications independently promote translational efficiency of the *SUP4* tRNA, we investigated whether *SUP4* defects associated with modification loss can be compensated by elevated eEF1A levels. We used a multicopy construct containing the *TEF1* gene (pTEF1)^53^ and introduced it into the *SUP4 elp3* strain defective in *ade2-1* ochre suppression. As shown in Fig. 4B, there is a clear restoration of growth on −Ade media of the *SUP4 elp3* strain, dependent on the *TEF1* expression construct. Also, read-through in the *lacZ*-UAA reporter is partially restored in *elp3* cells overexpressing eEF1A compared to the *elp3* strain without extra pTEF1. Thus, the functional defect in *SUP4* tRNA induced by loss of the mcm^5^U modification can be partially compensated by elevated eEF1A levels. To test whether this effect is unique to the mcm^5^U defective *elp3* strain or more general and extends to other modification genes, deletion of which also impairs *SUP4* nonsense suppressor efficiency, we analysed rescue by elevated eEF1A levels of *ade2-1* suppression in *pus7* and *ncl1* mutants. As shown in Fig. 5B, overexpression of *TEF1* indeed partially restored nonsense suppression in these mutant backgrounds, consistent with an ability of elevated eEF1A levels to compensate functional defects induced by various tRNA modification defects.

### Rescue of modification defects in non-suppressor tRNA by elevated eEF1A levels

Combined loss of mcm^5^/s^2^U and ψ38 was shown to induce severe growth defects that correlate with translational inefficiency of tRNA^Gln^_UUG_^30,54^. Since these defects were suppressed by elevated copy numbers of hypomodified tRNA^Gln^_UUG_ and this tRNA represents the single tRNA in yeast, which carries mcm^5^s^2^U and ψ38, simultaneous absence of these modifications apparently results in a severe loss of function of particularly this tRNA species. *TEF1/TEF2* are negative genetic interactors of several tRNA modification genes (Table S2) and elevated levels of eEF1A were shown in this study to rescue hypomodified *SUP4* (Figs 4B, 5B). Therefore, we investigated whether higher than normal levels of eEF1A can also rescue functional defects in natural, non-suppressor tRNAs.

**Figure 6:**
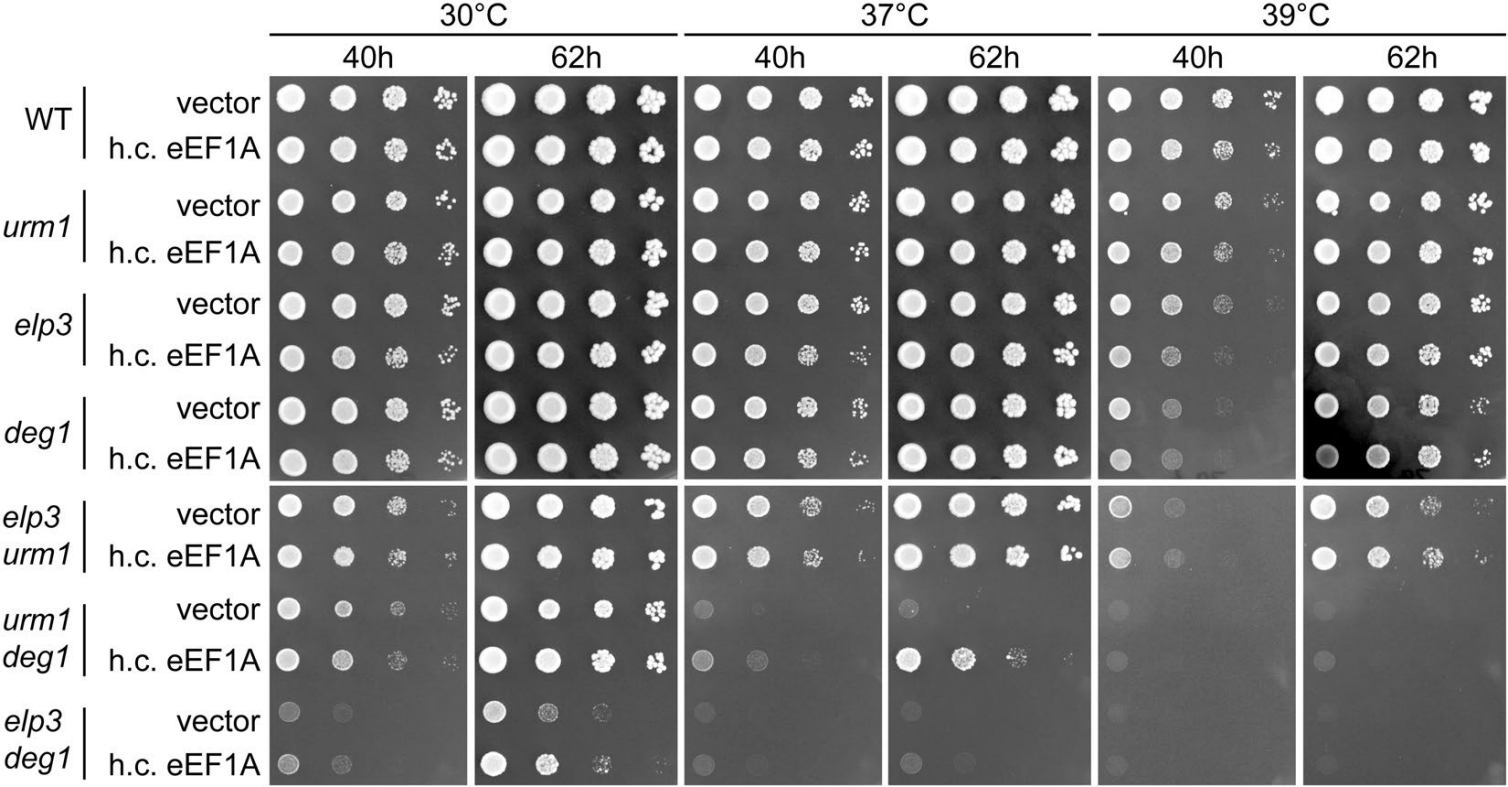
Heat sensitivity and growth defects in single and double tRNA modification mutants in the presence and absence of *TEF1* overexpression. h.c. eEF1A refers to overexpression of *TEF1* (pTEF1). Vector refers to YEplac181. Cells were taken from selective YNB medium and dilutions prepared in sterile water. After spotting of cell dilutions on YPD plates, these were incubated at 30 °C, 37 °C or 39 °C as indicated. Plates were photographed at the indicated time points.

To do so we tested whether growth defects in *deg1 urm1* (ψ38/s^2^U) or *deg1 elp3* (ψ38/mcm^5^U) under non-stressed or temperature stress (TS) conditions are ameliorated upon introduction of the *TEF1* multicopy construct (pTEF1) that rescued hypomodified *SUP4* (Fig. 5B). As shown in Fig. 6, there is indeed a clear improvement of growth of the *deg1 urm1* and *deg1 elp3* mutants, but not of the respective single mutants upon overexpression of eEF1A. Other than *deg1 elp3*, which is already severely impaired in growth under non-stressed conditions, the *deg1 urm1* mutant shows a severe growth defect only under conditions of mild heat stress (37 °C; Fig. 6). Growth of *deg1 elp3* under non-stressed conditions was partially rescued by pTEF1, whereas in *urm1 deg1*, pTEF1 was clearly beneficial under mild heat stress condition (Fig. 6). Rescue of the TS phenotype in *urm1 deg1* was only partial at 37 °C and undetectable at further elevated temperatures, suggesting that elevated eEF1A levels can improve functionality of hypomodified tRNA, but not to the level of fully modified tRNA. A sharp difference of growth responses between 37° and 39 °C was observed for several yeast strains defective in tRMA modification (Fig. S2)^30,54^. A partial phenotypic rescue at elevated temperature was also detected in a distinct double mutant (*tcd1 urm1*) lacking ct^6^A and s^2^U, in which tRNA^Lys^_UUU_ displays reduced functionality^30^ (Fig. S4), suggesting that functional defects in tRNA^Gln^_UUG_ and tRNA^Lys^_UUU_ in absence of critical modifications can be partly suppressed by elevating eEF1A abundance.

## Discussion

In this study, we quantified the functional relevance of all modifications within the extended ASL (including the VL) of a single tRNA species (*SUP4*) *in vivo*. Previous studies indicated a relevant contribution of ASL modifications mcm^5^U, ψ35 and i^6^A^10,34,35^. Indeed, all reporters used to measure *SUP4* efficiency identified loss of nonsense suppression in *elp3*, *pus7* and *mod5* mutants lacking these modifications individually. This result confirms previous studies of *elp3* and *mod5* mutants^10,34^ and provides first direct evidence for a crucial role of ψ35 in *SUP4* function. While it was shown that absence of an intron in the *SUP6* tRNA^Tyr^ derived ochre suppressor gene abolished both ψ35 formation and nonsense suppression^35^, a direct demonstration that the modification itself is important for suppressor tRNA function was lacking and is provided in the current study (Figs 1; 3). Since ψ can stabilize base pairs in RNA duplexes^55^, functional roles of the U to ψ isomerization at position 35 in *SUP4* could be to improve codon binding and competition against release factors. Consistent with Pus7 as the sole *in vivo* modifier for U35 in pre-tRNA^Tyr^^38^, we detected no functional relevance of the related Pus1 synthase in supporting nonsense suppression of *ade2-1* or *can1-100* by *SUP4*.

Classical reporters used to detect defects in nonsense suppression rely on the reversion of phenotypes linked to nonsense codons in genes causing auxotrophies or drug resistances. We find that *elp3*, *mod5* and *pus7* mutations confer adenine auxotrophy to a *SUP4 ade2-1 can1-100* strain and cause canavanine resistance, at first sight consistent with full loss of *SUP4* function. However, red pigmentation associated with *ADE2* gene defects is incompletely restored (Fig. 1) suggesting residual *SUP4* translational activity even in the absence of these modifications, which was already demonstrated for an *elp3* mutant using *lacZ* based reporter constructs^4,42^.

Although formally possible, we demonstrated that *SUP4* dependent suppression of *ade2-1* and *can1-100* does not involve an inhibition of NMD. First, inhibition of NMD by deletion of *UPF1* in the absence of *SUP4* suppressor tRNA did not revert *ade2-1* or *can1-100* phenotypes under the conditions tested (Fig. 2). Second, *SUP4* clearly affects *ade2-1* and *can1-100* phenotypes in the absence of NMD, ruling out the option that *SUP4* functions mainly via NMD inhibition. However, the restoration of *SUP4* dependent *ade2-1* and *can1-100* suppression in the absence of *ELP3* by inactivation of NMD (Fig. 2) also indicates that changes in reporter mRNA levels can affect total *SUP4* dependent nonsense read-through.

To quantify the effect of modification defects on *SUP4* function independent of potential mRNA fluctuations, we utilized dual luciferase reporter constructs^49^ and measured residual *SUP4* function in modification mutants. These constructs utilize an internal normalization of luciferase activity generated after nonsense read-through to an upstream reporter expressed independently of nonsense suppression and are therefore insensitive to changes of reporter mRNA levels. As expected, *mod5*, *pus7* and *elp3* mutations significantly reduced *SUP4* dependent nonsense suppression in this reporter without entirely abolishing it. In addition, *ncl1* and *dus3* mutations (lack of m^5^C or D47) reduced *SUP4* functions significantly. The *ncl1* effect correlated with a full loss of *can1-100* suppression and a partial defect in *ade2-1* suppression, while the *dus3* mutation did not affect these reporters and lowered dual luciferase based *SUP4* read-out less drastically compared to *ncl1*, *elp3*, *mod5* or *pus7* mutations.

In tRNA^Tyr^, the Ncl1 dependent m^5^C is present at position 48, which forms a non-canonical basepair (Levitt basepair) with the nucleoside in position 15 during tRNA folding^2,56^. However, multiple tRNAs naturally lack this modification and form the Levitt pair without m^5^C modification^2,36^. Therefore, the contribution of the m^5^C modification to tRNA stability and function remains obscure. To our knowledge, this study provides first *in vivo* evidence for a clear positive role of m^5^C48 modification in maintaining function of a defined tRNA. While absence of Ncl1 is tolerated without severe phenotypes in yeast (Fig. S2), mutation of the human *NCL1* orthologue NSUN2 is linked to intellectual disability, like several other mutations affecting tRNA modifiers^26,57^. Thus, m^5^C likely fulfils important functions in yeast and human tRNAs.

In contrast to anticodon loop modifications and m^5^C, neither m^2,2^G nor ψ39 influenced *SUP4* function significantly in any of the reporters used. Hence, there is a differential importance of individual modifications for *SUP4* function. While all the anticodon loop modifications are important on their own, stem and VL modifications can be either dispensable (m^2,2^G, ψ39) or important (m^5^C, D47). The dispensability of ψ39 for *SUP4* function was also observed using a *lacZ* based reporter^42^ and provides an example for an extreme position effect of modification importance: Pus7 dependent ψ within the anticodon loop (position 35) is crucial, whereas Deg1 dependent introduction of the same modification in the anticodon stem (position 39) is much less important. Since ψ is thought to contribute rigidity and reduce flexibility of the sugar phosphate backbone^58^, its presence in the loop might be of higher functional relevance compared to the stem location due to a potential contribution to stabilizing the open loop configuration. Indeed, in the *sup70–65* amber suppressor derived from tRNA^Gln^_CUG_, in which a Deg1 dependent ψ is in position 38 (loop), this modification is crucial for stop codon read-through^42,59^. However, not only ψ35 but also ψ39 appears to be conserved within tRNA^Tyr^ as a large majority of sequenced eukaryotic tRNA^Tyr^ species contain this modification or one of its derivatives (1-methylpseudouridine, 2′-O-methylpseudouridine)^36^. Thus, despite the absence of effects on *SUP4* upon its removal alone, a subtler contribution of ψ39 to *SUP4* function appears likely.

One possibility to explain absence of effects in single mutant backgrounds is functional redundancy with other modifications. In such case, effects would only be observable in combined absence with modifications that contribute to the same functional aspect. Indeed, our double mutant approach revealed a reduction of *SUP4* function upon removal of ψ39 when the tRNA was already missing any of the extended ASL modifications, except for mcm^5^U which could not be tested due to synthetic lethality (Fig. 3). Due to its position in the anticodon stem, ψ39 might improve RNA duplex stability which may become critical only after additional modification defects that destabilize tRNA by other means. A similar trend was observed for Trm1 dependent m^2,2^G, which was dispensable alone but absence of which clearly reduced *SUP4* function when this tRNA was already lacking any of the anticodon loop modifications. m^2,2^G contributes to tRNA folding by preventing base pairing within the internal loop but this contribution is not essential, since several tRNAs lack this modification and *trm1* mutation is tolerated without severe phenotypes in yeast^2,36^ (Fig. S2). Again, upon introduction of additional modification defects that may weaken structural stability of the tRNA via a distinct mechanism, more drastic functional defects might be induced. Such effect was most pronounced for the combination of i^6^A and m^2,2^G defects, which had a strong synergistic negative effect on *SUP4* function (Figs 3, S3). In general, absence of i^6^A amplified the negative effects induced by most additional modification defects, and combination of i^6^A and mcm^5^U deficiencies nearly entirely eliminated residual *SUP4* function (Fig. 3). A37 is at risk of pairing with the conserved U33, which results in destabilization of the anticodon loop and which is why A37 is often modified by bulky side chains (i^6^A, ct^6^A, yW) occupying the Watson-Crick interface^1,6,36^. Absence of i^6^A37 modification therefore likely has a destabilizing effect on the anticodon loop which may sensitize tRNA to loss of other modifications (such as m^2,2^G) that would further contribute to tRNA structure and stability. Since mcm^5^s^2^U was previously identified to contribute to tRNA^Lys^_UUU_ anticodon loop prestructuring along with the A37 modification 2-methylthio-N6-threonylcarbamoyladenosine^6^, it appears very likely that a similar functional collaboration exists between the mcm^5^U and i^6^A modifications in the *SUP4* tRNA.

Our study further indicates that eEF1A abundance constitutes an independent factor which modulates the UAA read-through capacity of the *SUP4* suppressor tRNA. In the presence of all modifications, *SUP4* efficiency is clearly decreased upon reducing eEF1A gene dosage from its natural two copies (*TEF1/TEF2*) to one (*tef1/TEF2*) (Figs 4A,C, 5A). This negative effect is additive with a partial loss of *SUP4* function caused by the absence of the m^5^C modification (Fig. 5A). While m^5^C deficient *SUP4* is still able to mediate partial suppression of *ade2-1*, this property is lost upon additional deletion of one of the two eEF1A encoding genes. This result indicates that tRNA modifications which are of minor functional relevance under standard conditions, can become rather critical under conditions of reduced eEF1A availability. One prediction of such contribution of eEF1A abundance to tRNA efficiency would be that functional impairments by loss of critical modifications could potentially be compensated by elevating eEF1A levels. Indeed, our results support this notion, since *ade2-1* nonsense suppression defects of *SUP4* tRNA lacking either mcm^5^U, ψ35 or m^5^C can be significantly rescued by overexpression of eEF1A (Figs 4B, 5B). In addition, the TS phenotype caused by dual modification loss in two natural non-suppressor tRNA species, tRNA^Gln^_UUG_ or tRNA^Lys^_UUU_, can be partially suppressed by overexpression of eEF1A (Figs 6, S4). Interestingly, a similar TS phenotype caused by a defect of tRNA^Phe^ in *trm7* mutants was recently shown to be partially suppressible by an additional copy of *TEF1/2*^51^. Thus, the positive effect of increasing eEF1A abundance on tRNA function *in vivo* extends from nonsense suppressor tRNA to at least three different natural tRNAs lacking critical modifications. Together our results indicate that tRNA modifications and eEF1A abundance constitute factors that appear to independently control the translational efficiency of tRNAs. If both are impaired, additive negative effects can be demonstrated, whereas elevated levels of eEF1A might generally compensate for defects caused under conditions of inappropriate tRNA modifications.

## Methods

### General methods

Yeast strains used in this study are listed in Table S1 and were cultivated in yeast peptone dextrose (YPD) or yeast nitrogen base minimal medium lacking specific nutrients to select for gene deletions or plasmids^60^. For targeted gene deletion, PCR products containing auxotrophic marker genes (*KlLEU2* or *SpHIS5*) with flanking regions of 50 nucleotides homology to target genes were generated according to^61^ and transformed into yeast strains using the lithium acetate method^62^. Correct target gene replacement was verified using PCR and involved marker gene specific oligonucleotides in combination with primers binding the target locus outside of the region covered by the gene deletion PCR product. A plasmid shuffle assay to check for viability of an *elp3 deg1* double mutant involved pFF8 (CEN-*ELP3-URA3*)^63^ and subsequent counterselection on 5-FOA containing medium as described earlier^30^.

### Phenotypic growth and nonsense suppression assays

Yeast strains were grown on YPD or leucine free YNB solid media to select for the presence of plasmids YEp181 or pTEF1 (multi copy *TEF1*)^53^ for 2 to 3 days. Subsequently, cells were recovered from the plates and resuspended in sterile water and dilutions prepared with OD600nm values of 0.15, 0.015, 0.0015 and 0.00015. These were spotted on either solid YPD medium or YNB medium lacking adenine or lacking arginine and containing canavanine sulfate (60 µg/ml). Subsequently, plates were incubated at indicated temperatures for 30–90 h and photographed at indicated time points.

### Beta galactosidase assay

Yeast strains transformed with pUKC815 (wild type *lacZ*) or pUKC817 (UAA ochre insertion in *lacZ*)^52^ were grown in uracil free YNB medium to OD600 nm of 2–3. Cells were washed once with Z-buffer (60 mM Na_2_HPO_4_, 40 mM NaH_2_PO_4_, 10 mM KCl, 50 mM 2-mercaptoethanol, pH 7) and subsequently resuspended using the same buffer. Cell density at 600 nm was measured and 500 µL aliquots transferred to new tubes. To each, two drops of 0.01% sodium dodecyl sulphate (SDS) solution and chloroform were added, followed by vigorous mixing and incubation at 37 °C for 5 min. Reactions were started by addition of 100 µL of 4 mg·mL^−1^ ortho-nitrophenyl-β-galactoside dissolved in Z-buffer and stopped by the addition of 250 µL 1 M Na_2_CO_3_. Activity units were calculated using OD420 nm absorbance of samples by employing Miller’s formula^64^. Relative read-through efficiency (%) was determined using the ratio of the beta galactosidase activities measured with the pUKC817 and pUKC815 constructs. For each strain, at least three independent cultures were measured with both constructs. Significant differences of *SUP4* activity in the wild type and mutant strains or between the *elp3* mutant with and without eEF1A overexpression were determined using two-tailed t-test as described before for yeast beta galactosidase reporter assays^23^.

### Dual luciferase assay

Dual luciferase assays were performed using the dual luciferase reporter (DLR) assay kit (Promega). Yeast strains of the W303-1B background with or without *SUP4* were transformed separately with two different dual luciferase reporter plasmids^49^, the reporter construct containing a UAA nonsense codon (pDB723) or the control construct (pDB688) containing a sense codon at the same position between the renilla and firefly luciferase genes. Strains were grown in uracil free YNB medium and assayed for luminescence with a Promega Glomax luminometer as described^49^. The relative read-through level (read-through %) was calculated using the firefly-renilla luciferase activity (nonsense) ratio divided by the firefly-renilla luciferase activity (sense) ratio multiplied by 100. At least four independent transformants for each strain and construct were measured. Significant differences of *SUP4* activity in the absence and presence of modification defects were determined by two-tailed t-test as described before for dual luciferase assays in yeast^23^.

## Electronic supplementary material

Supplementary information

Supplementary Dataset 1

Supplementary Dataset 2

Supplementary Dataset 3
