# Supplementary material for: Collaboration of tRNA modifications and elongation factor eEF1A in decoding and nonsense suppression
Source: Sci Rep. 2018 Aug 24;8:12749. doi: 10.1038/s41598-018-31158-2 (PMC6109124; doi:10.1038/s41598-018-31158-2)
Supplement: Supplementary file 1 — Supplementary information [file 41598_2018_31158_MOESM1_ESM.docx]

**Collaboration of tRNA modifications and elongation factor eEF1A in decoding and nonsense suppression**

Roland Klassen and Raffael Schaffrath

Institut für Biologie, Fachgebiet Mikrobiologie, Universität Kassel, Heinrich-Plett-Str. 40, D-34132 Kassel, Germany

**Supplementary information**

Table S1: Strains used in this study

| Strain | Genotype | Reference/source |
| --- | --- | --- |
| *S. cerevisiae* BY4741 | MATa, *his3Δ, leu2Δ, met15Δ, ura3Δ* | Euroscarf, Frankfurt |
| *S. cerevisiae* elp3 | BY4741 *elp3ΔKanMX4* | Euroscarf, Frankfurt |
| *S. cerevisiae* urm1 | BY4741 *urm1ΔKanMX4* | Euroscarf, Frankfurt |
| *S. cerevisiae* deg1 | BY4741 *deg1ΔKanMX4* | Euroscarf, Frankfurt |
| *S. cerevisiae* tcd1 | BY4741 *tcd1ΔKanMX4* | Euroscarf, Frankfurt |
| *S. cerevisiae* elp3 urm1 | BY4741 *elp3ΔKanMX4 urm1ΔHIS3* | ^1^ |
| *S. cerevisiae* deg1 urm1 | BY4741 *urm1ΔKanMX4 deg1ΔSpHIS5* | ^2^ |
| *S. cerevisiae* deg1 elp3 | BY4741 *elp3ΔKanMX4 deg1ΔSpHIS5* | ^2^ |
| *S. cerevisiae* tcd1 urm1 | BY4741 *tcd1ΔKanMX4 urm1ΔSpHIS5* | ^2^ |
| *S. cerevisiae* W303-1B | MATα *leu2-3,112 trp1-1 can1-100 ura3-1 ade2-1 his3-11,15* | ^3^ |
| *S. cerevisiae* UMY2893 | MATα *SUP4* *leu2-3,112 trp1-1 can1-100 ura3-1 ade2-1 his3-11,15* |  |
| *S. cerevisiae* UMY2916 | UMY2893 *elp3∆KanMX4* | ^4^ |
| *S. cerevisiae* RK260 | UMY2893 *tef1ΔSpHIS5* | this work |
| *S. cerevisiae* RK208 | UMY2893 *deg1ΔSpHIS5* | ^5^ |
| *S. cerevisiae* RK402 | UMY2893 *mod5ΔSpHIS5* | this work |
| *S. cerevisiae* RK403 | UMY2893 *pus1ΔSpHIS5* | this work |
| *S. cerevisiae* RK404 | UMY2893 *elp3∆KanMX4 mod5ΔSpHIS5* | this work |
| *S. cerevisiae* RK421 | UMY2893 *ncl1ΔSpHIS5* | this work |
| *S. cerevisiae* RK424 | UMY2893 *ncl1ΔSpHIS5 tef1ΔKlLEU2* | this work |
| *S. cerevisiae* RK427 | UMY2893 *mod5ΔSpHIS5 ncl1ΔKlLEU2* | this work |
| *S. cerevisiae* RK428 | UMY2893 *pus7ΔSpHIS5* | this work |
| *S. cerevisiae* RK431 | UMY2893 *mod5ΔSpHIS5 deg11ΔKlLEU2* | this work |
| *S. cerevisiae* RK432 | UMY2893 *pus7ΔSpHIS5 ncl1ΔKlLEU2* | this work |
| *S. cerevisiae* RK436 | UMY2893 *elp3ΔKanMX4 pus7ΔKlLEU2* | this work |
| *S. cerevisiae* RK437 | UMY2893 *deg1ΔSpHIS5 pus7ΔKlLEU2* | this work |
| *S. cerevisiae* RK441 | UMY2893 *mod5ΔSpHIS5 pus7ΔKlLEU2* | this work |
| *S. cerevisiae* RK444 | UMY2893 *ncl1ΔSpHIS5 elp3ΔKlLEU2* | this work |
| *S. cerevisiae* RK446 | UMY2893 *elp3ΔKanMX trm1ΔKlLEU2* | this work |
| *S. cerevisiae* RK447 | UMY2893 *pus7ΔSpHIS5 trm1ΔKlLEU2* | this work |
| *S. cerevisiae* RK448 | UMY2893 *mod5ΔSpHIS5 trm1ΔKlLEU2* | this work |
| *S. cerevisiae* RK449 | UMY2893 *pus7ΔSpHIS5 dus3ΔKlLEU2* | this work |
| *S. cerevisiae* RK450 | UMY2893 *trm1ΔKlLEU2* | this work |
| *S. cerevisiae* RK451 | UMY2893 *dus3ΔKlLEU2* | this work |
| *S. cerevisiae* RK453 | UMY2893 *dus3ΔKlLEU2 elp3ΔSpHIS5* | this work |
| *S. cerevisiae* RK454 | UMY2893 *dus3ΔKlLEU2 trm1ΔSpHIS5* | this work |
| *S. cerevisiae* RK455 | UMY2893 *ncl1ΔSpHIS5 trm1ΔSpHIS5* | this work |
| *S. cerevisiae* RK456 | UMY2893 *deg1ΔSpHIS5 ncl1ΔKlLEU2* | this work |
| *S. cerevisiae* RK457 | UMY2893 *ncl1ΔSpHIS5 dus3ΔKlLEU2* | this work |
| *S. cerevisiae* RK458 | UMY2893 *deg1ΔSpHIS5 trm1ΔKlLEU2* | this work |
| *S. cerevisiae* RK459 | UMY2893 *deg1ΔSpHIS5 dus3ΔKlLEU2* | this work |
| *S. cerevisiae* RK460 | UMY2893 *mod5ΔSpHIS5 trm1ΔKlLEU2* | this work |
| *S. cerevisiae* RK461 | UMY2893 *mod5ΔSpHIS5 dus3ΔKlLEU2* | this work |
| *S. cerevisiae* RK503 | W303-1B *upf1ΔKlLEU2* | this work |
| *S. cerevisiae* RK504 | UMY2893 *upf1ΔKlLEU2* | this work |
| *S. cerevisiae* RK505 | UMY2893 *elp3ΔKanMX4 upf1ΔKlLEU2* | this work |

Table S2: Negative genetic interactors of *TEF1*  ^6^. File Table S2.xlsx

Table S3: Negative genetic interactors of *TEF2* ^6^. File Table S3.xlsx

Table S4: Gene ontology analysis of negative *TEF1* and *TEF2* interactors. File Table S4.xlsx

**
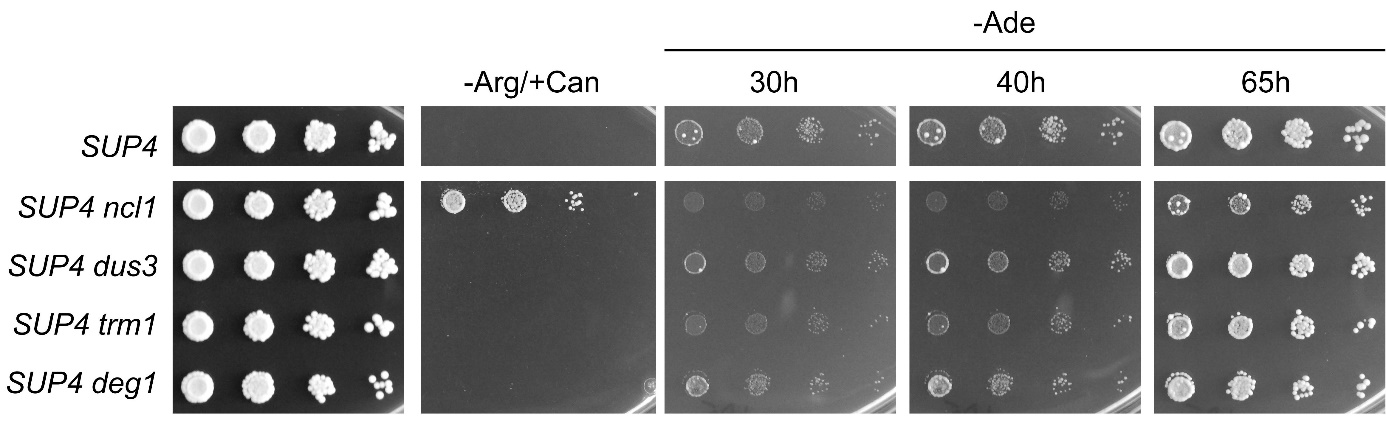
**

Figure S1: *SUP4* mediated nonsense suppression in additional single mutants. Indicated strains were spotted on either YPD, -Arg/+Can or -Ade. YPD ad -Arg/+Can plates were incubated for 48 h, -Ade plates for indicated time periods.

**
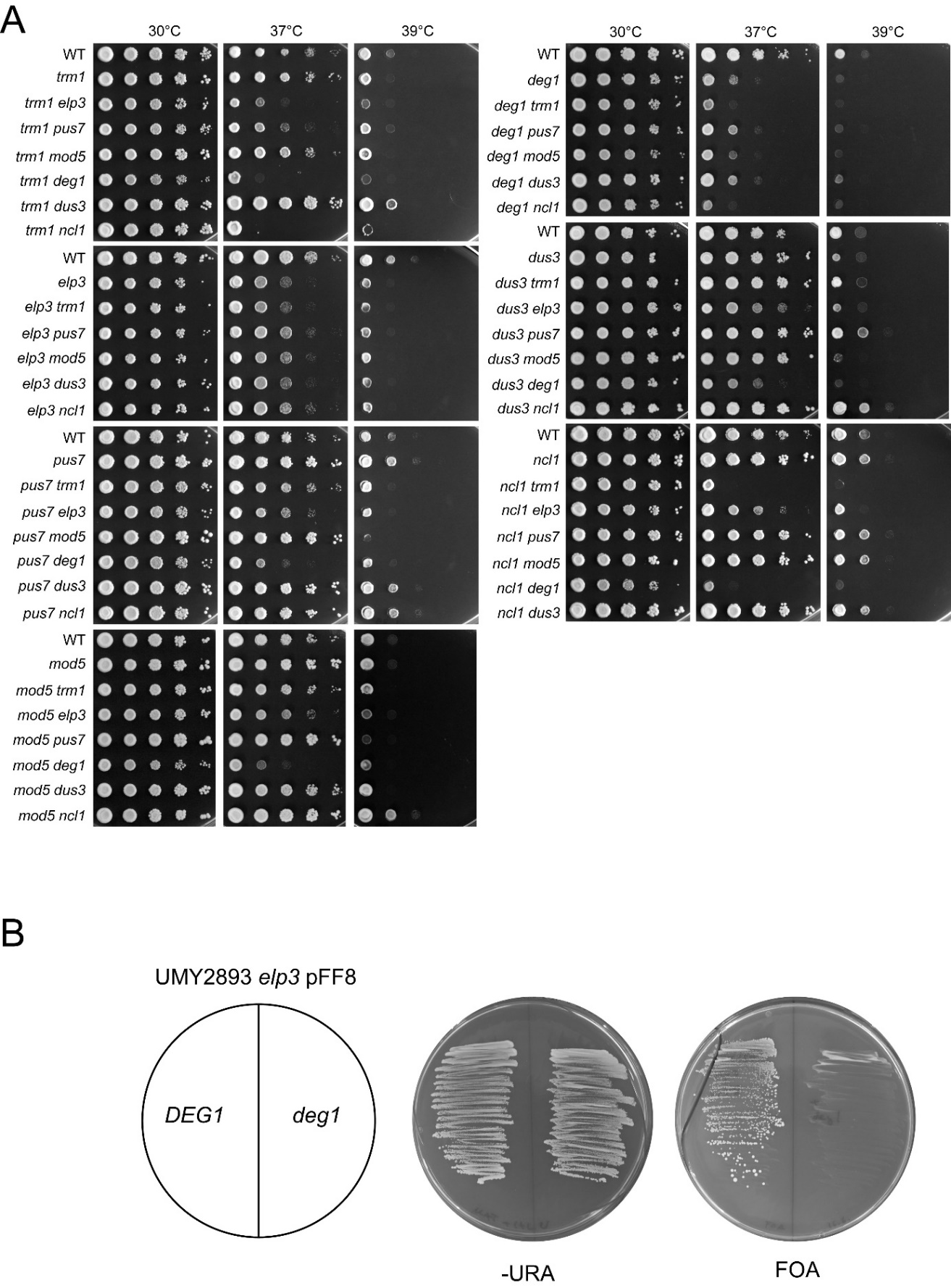
**

Figure S2: Construction and initial phenotypic analysis of tRNA modification mutants in the *SUP4* strain. (A) Plate assay of single and double tRNA modification mutants for growth defects at 30, 37 or 39°C. (B) Plasmid shuffle assay to study viability of *SUP4 elp3 deg1*. The *SUP4 elp3* strain was transformed with pFF8 (CEN-*URA3-ELP3*) and subsequently *DEG1* deleted. The resulting strain was checked for viability on FOA (counterselection of pFF8) as detailed in the Methods section under ’general methods’.

**
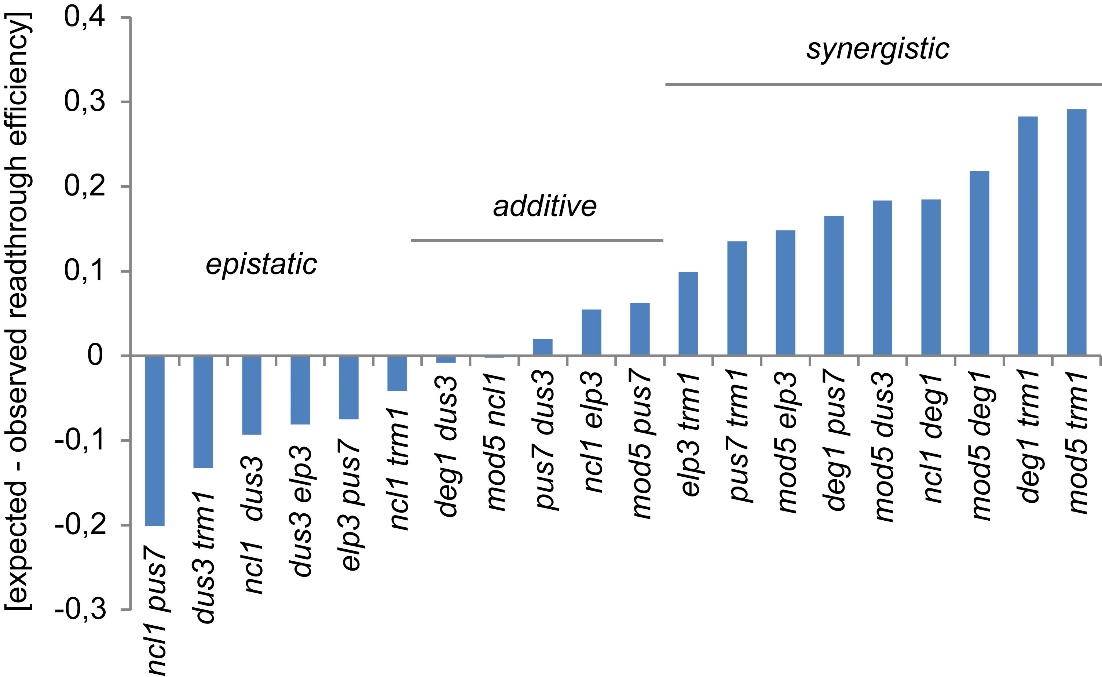
**

Figure S3: Epistasis of tRNA modification defects in *SUP4* translational efficiency. Epistasis score was calculated as described in the results section and mutants sorted according to their respective value. Strong synergistic negative effects on *SUP4* efficiency result in positive epistasis scores.

**
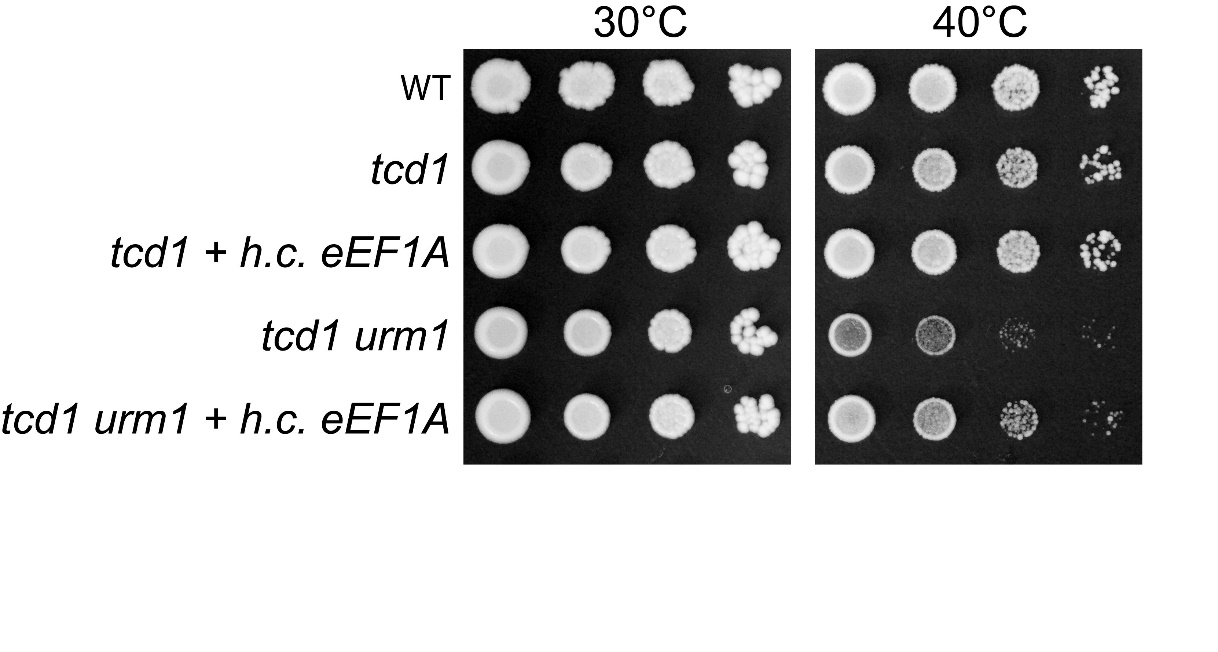
**

Figure S4: Rescue of growth defects in *tcd1 urm1* and *tcd1 elp3* mutants by overexpression of *TEF1* (pTEF1). Assay was conducted as detailed in Fig.6.

**References**

1. Klassen, R. *et al.* Loss of anticodon wobble uridine modifications affects tRNA(Lys) function and protein levels in Saccharomyces cerevisiae. *PloS one* **10,** e0119261 (2015).

2. Klassen, R. *et al.* tRNA anticodon loop modifications ensure protein homeostasis and cell morphogenesis in yeast. *Nucleic acids research* **44,** 10946–10959 (2016).

3. Fiorentini, P., Huang, K. N., Tishkoff, D. X., Kolodner, R. D. & Symington, L. S. Exonuclease I of Saccharomyces cerevisiae functions in mitotic recombination in vivo and in vitro. *Molecular and cellular biology* **17,** 2764–2773 (1997).

4. Huang, B., Johansson, M. J. O. & Byström, A. S. An early step in wobble uridine tRNA modification requires the Elongator complex. *RNA (New York, N.Y.)* **11,** 424–436 (2005).

5. Klassen, R. & Schaffrath, R. Role of Pseudouridine Formation by Deg1 for Functionality of Two Glutamine Isoacceptor tRNAs. *Biomolecules* **7** (2017).

6. Costanzo, M. *et al.* A global genetic interaction network maps a wiring diagram of cellular function. *Science (New York, N.Y.)* **353** (2016).
